# Prevalence of Mistreatment or Belittlement among Medical Students – A Cross Sectional Survey at a Private Medical School in Karachi, Pakistan

**DOI:** 10.1371/journal.pone.0013429

**Published:** 2010-10-15

**Authors:** Sana Shoukat, Mariam Anis, Danesh K. Kella, Fahad Qazi, Fatima Samad, Faizia Mir, Maryah Mansoor, Mohammad B. Parvez, Bushra Osmani, Sukaina A. Panju, Haider Naqvi

**Affiliations:** Department of Psychiatry, Aga Khan University, Karachi, Pakistan; Aga Khan University Karachi, Pakistan

## Abstract

**Background:**

Mistreatment or belittlement of medical students either by faculty or fellow students has often been reported. Perception of mistreatment has also been associated with increased degree of psychological morbidity. There is a lack of such studies being conducted amongst the medical students of Pakistan. The aim of this study was to determine the prevalence and forms of perceived mistreatment and presence of mental health morbidity in a private medical school in Pakistan. Also, any association between mental health morbidity and mistreatment was to be identified.

**Methods:**

A cross sectional study was carried out on medical students from Aga Khan University Hospital, Karachi, Pakistan during the period of June–September 2007. A self administered questionnaire, adapted from Frank et al and Baldwin et al was distributed to a total of 350 students. The questionnaire consisted of three parts: the first dealing with the demographics of the population, the second concerning the various forms of mistreatment, while the third assessed the mental health of students using the General Health Questionnaire 12(GHQ12). Descriptive statistics were performed. The Chi-square test and Fisher's exact tests were applied.

**Results:**

A total of 350 students were approached out of which 232 completed the questionnaire giving a response rate of 66.2%. Mistreatment was reported by 62.5% (145/232) of the respondents. Of these, 69.7% (83/145) were males and 54.9% (62/145) were females. There was a significant relationship between gender, year division, stress at medical school and possible use of drugs/alcohol and reported mistreatment but no statistical relationship was seen with psychiatric morbidity. The overall prevalence of psychological morbidity was 34.8% (77/221).

**Conclusion:**

This study suggests high prevalence of perceived mistreatment and psychological morbidity among Pakistani medical students. However, no association was found between these two aspects of medical student education. There is a need to bring about changes to make the medical education environment conducive to learning. Increased student feedback, support systems and guidance about progress throughout the year and the provision of adequate learning resources may provide help with resolving both of these issues.

## Introduction

Mistreatment occurs at both personal and organizational levels in schools [1], workplaces [2], [3] and educational institutions. It has been seen to take various forms including belittlement, harassment, discrimination, threats, assaults and discouragement [4].

However, when found in medical schools, it has been overlooked and partly accepted as part of the physician career training. A large number of studies have focused on mistreatment and harassment of medical students all over the world [4], [5], [6], [7], [8], [9], [10]. These studies include a U.S national survey showing the perception of harassment and belittlement to be as high as 42% and 84% respectively [5]. They also indicate a negative impact of mistreatment on student mental health and satisfaction with careers. Hence, where medical institutions have been aiming to instill positive professional identities, negative influences have been enforced due to prevailing ill-treatment. Such negative influences adopted at university life are seen to continue and create hostile learning and working environments.

Despite having a better health profile in other aspects, medical students have been seen to have a higher prevalence of psychiatric morbidity than the general population or when compared to other university students [11], [12], [13], [14], [15]. More importantly, development of psychiatric illness later has been seen to be strongly associated with perceived medical school stress [16].

Medical student mistreatment and abuse has remained an unexplored area in Pakistan. At the same time, local figures for psychiatric morbidity are as high as 60–70% [17], [18]. Hence, we aimed to determine the prevalence and forms of perceived mistreatment and presence of mental health morbidity in a private medical school in Pakistan. Also, any association between mental health morbidity and mistreatment was to be identified.

## Methods

A cross sectional study was carried out on medical students from Aga khan University Hospital, Karachi, Pakistan during the period of June–September 2007. Permission to perform the survey was obtained from the Chair, Department of Psychiatry of the university. Informed Consent was taken by all the students on paper. Respondents were assured of utmost confidentiality.

Medical students from all five years were included in the study. Exclusion was based on refusal to the informed consent. Data was collected during working hours from the libraries, computer labs, courtyard and cafeteria. All students were approached from the five classes of the institution.

The research tool was a self administered questionnaire, adapted from Frank et al and Baldwin et al. [4], [5] Minimal modification was done so that the results remain comparable to the other studies conducted. It contained three parts: the first part (15 questions) based on students demographics including age, sex, religion, marital status, ethnicity and background, schooling and education, year of medical school, having a physician parent or a close relative, monthly household income and current interest in specialty which was to be pursued in future.

The second part comprised of 29 questions structured to gather information regarding frequency with which the respondents had experienced different types of perceived mistreatment or harassment over the course of medical school as well as the source of that perceived mistreatment. Items included being shouted or yelled at, being belittled or humiliated, being assigned task for punishment rather than educational value, having someone take credit for respondents work, being physical threatened, hit, slapped, kicked or pushed and being threatened with an unfair grade as well experiencing sexual harassment or exploitation and racial, ethnic or gender discrimination. For each item respondents were asked to indicate how often this experience had happened specifically to them: Never, Sometimes, and Often. The students were asked to indicate the source of each type of perceived mistreatment from a list that included Residents, Clinical Faculty, Nurses, Classmate/Seniors and Basic Science faculty. Additional questions about satisfaction with career, the medical institution and the faculty as well as perception of use of substance and alcohol to cope were also included where respondents chose to strongly disagree, remain neutral or strongly agree to statements.

The third part of the questionnaire dealt with assessment of mental health of medical students using the 12 item- General Health Questionnaire12. The 12 item General Health Questionnaire is a self administered screening instrument designed to detect current, diagnosable psychiatric distress. It mainly covers four identified elements of distress:

- Depression
- Anxiety
- Social impairment
- Hypochondriasis

The responses in the questionnaire are described as; “much less than usual”, “same as usual”, “more than usual”, and “much more than usual”. The standard scoring method recommended by Goldberg for the need of case identification is called “GHQ method” also known as the binary method. In this method the two least symptomatic answers score 0 and the two most symptomatic answers score 1 – i.e. 0-0-1-1. The minimum GHQ-12 total score was 0 and the maximum GHQ-12 total score was 12.

The cut off for presence of psychiatric morbidity was taken as 3 with the tool having the following validation coefficients at this cutoff [19], [20], [21].

- Reliability 0.85 (as seen by the Cronbach's alpha)
- Sensitivity 52–81.3%
- specificity 74–85%
- overall misclassification rate 30

The data was entered in Epi Info and analyzed using SPSS (Statistical Package for Social Sciences, version 16.0).

Data was entered in Epi Info and analyzed in Statistical Package for Social Sciences version 16.0 (SPSS, Inc., Chicago, IL, USA). Descriptive statistics were performed. Results were recorded as frequencies, means ± standard deviations (SD) and p-values. The Chi-square test and Fisher's exact test were used for univariate analysis of categorical variables. Tables and figures were used for viewing of the results. A p-value of <0.05 was taken as significant for all purposes.

## Results

A total of 350 students were approached out of which 232 completed the questionnaire giving a response rate of 66.2%. The mean age of the respondents was 21.40±1.75 years. There were 119(51.3%) males and 113(48.7%) females among the respondents. The demographics of the respondents are listed in Table 1.

**Table 1:** Demographics of the study population.

	% (n)	
**Gender**		
Male	**51.3 (119)**	
Female	**48.7 (113)**	
**Religion**		
Islam	**95.7 (222)**	
Christanity	1.7	**(4)**
Hindusim	1.3	**(3)**
Other	1.3	**(3)**
**Marital Status**		
Single	95.7	**(222)**
Married	4.3	**(10)**
**Relative Doctor**		
Yes	56.0	**(130)**
No	44.0	**(102)**
**Ethinicity**		
Punjabi	42.7	**(99)**
Pathan	13.8	**(32)**
Sindhi	6.9	**(16)**
Balouchi	1.7	**(4)**
Urdu Speaking	21.1	**(49)**
Other	13.8	**(32)**
**Background**		
Rural	22.0	**(51)**
Urban	78.0	**(181)**
**Year of medical School**		
1st year	19.4	**(45)**
2nd year	11.6	**(27)**
3rd year	23.3	**(54)**
4th year	25.9	**(60)**
5th year	19.8	**(46)**
**Residence**		
Hostelite	56.9	**(132)**
Day Scholar	43.1	**(100)**
**Monthly household income**		
10,000–50,000	22.4	**(52)**
50,000–100,000	36.6	**(85)**
>100,000	36.2	**(84)**
Did not report	4.7	**(11)**
**Education System**		
Intermediate	33.2	**(77)**
A-levels	62.9	**(146)**
Other	3.9	**(9)**
**Failed continuous Assessment**		
Yes	13.4	**(31)**
No	82.3	**(191)**
Did not report	**4.3**	**(10)**

**Figure 1:**
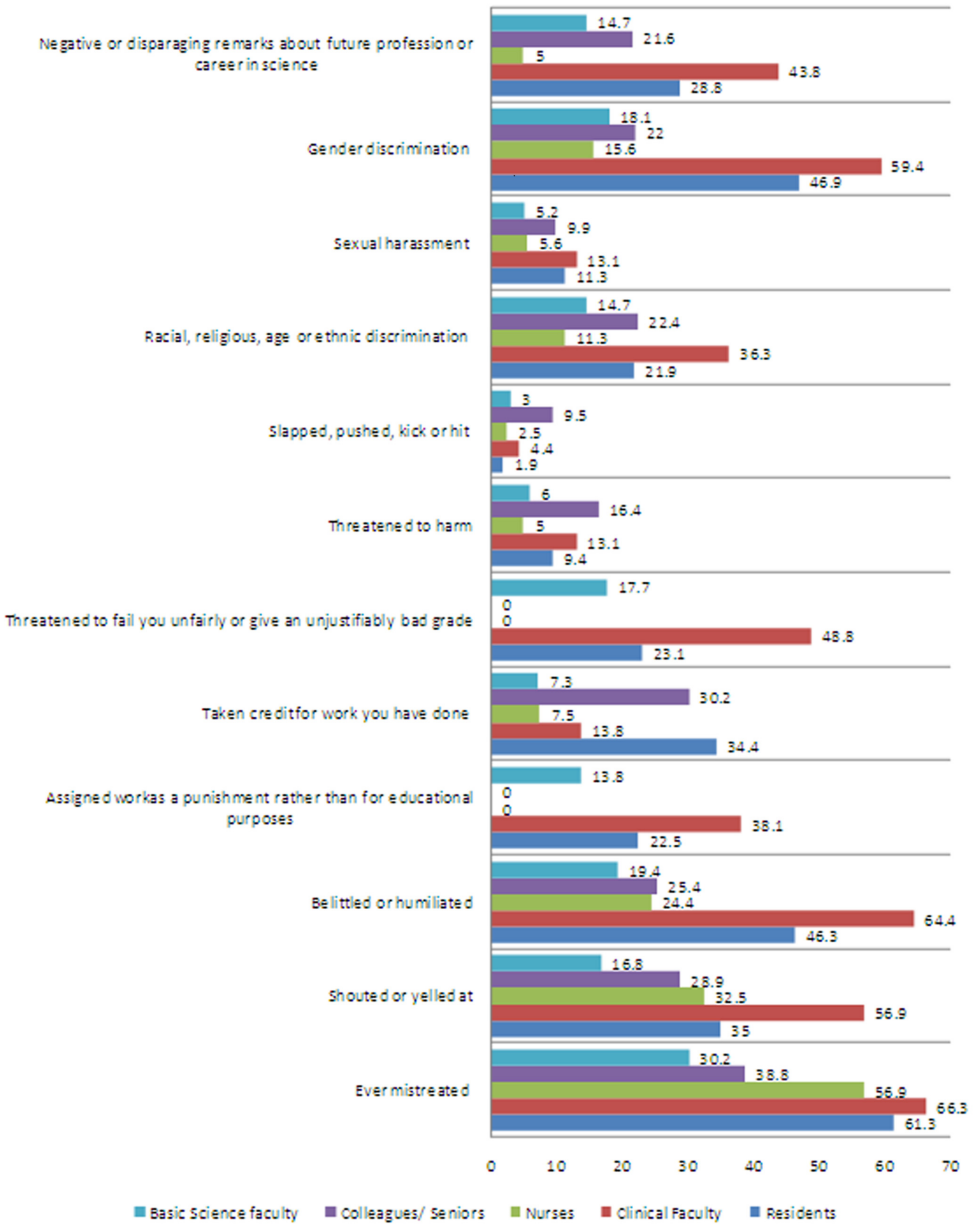
Types of mistreatment reported by medical students.

Mistreatment was reported to be 62.5% (145/232) by medical students. Of these, 69.7% (83/145) were males and 54.9% (62/145) were females. Males were more likely to have reported mistreatment as compared to females (p = 0.019). There was significant relationship between gender, year division, stress at medical school and possible use of drugs/alcohol and reported mistreatment but no statistical relationship was seen with psychiatric morbidity (See Table S1). The part dealing wit stress had three major components including the medical school trying to reduce stress, the medical school having good coping strategies to eliminate stress and lastly the use of drugs or smoking by students for reducing stress. Amongst these all were significant except for the use of smoking as a coping strategy. Students did report the use of alcohol for reducing stress. Failing a continuous assessment was also reported to be significant. Other factors including religion, relative being a doctor, ethnicity, marital status and background and was not significantly related to the reported mistreatment. The various forms of mistreatment reported by basic science faculty, colleagues/seniors, nurses, clinical faculty and residents is shown in Fig 1. The greatest proportion of students reported to have been mistreated by clinical faculty 67.5% (108/160). Racial or religious discrimination, belittled or humiliated, shouted or yelled at, assigned extra tasks as punishment, threatened to fail or grade unfairly, sexual harassment, gender discrimination and negative or disparaging remarks about future career were most commonly reported to have occurred from the clinical faculty. Compared to this residents were most commonly reported for taking credit for work that the student's had done. Physical assault came mainly from fellow students.

The mean GHQ score of the study population was 2.93±2.95. The prevalence of psychiatric morbidity as determined by GHQ score of greater than 3 was 34.8% (77/221).These students were also found to be complaining that the medical school doesn't have good stress coping mechanisms. (Table S2)

## Discussion

Much research has been conducted in the past to assess the prevalence of mistreatment of medical students including cross-sectional, longitudinal surveys and descriptive analyses [1], [5], [10], [22], [23]. Similar to other studies conducted across the world, we report in our study, a high prevalence of perceived mistreatment among medical students [22], [23], [24]. However, even similar numbers have a different meaning in these two settings as medical students in Pakistan tend to be undergraduates, younger and have a longer stay in medical school as compared to their international counterparts as they are enrolled into a five year medical program as compared to a four year program internationally. Other cultural, educational, and contextual differences should also be accounted for when comparing the results of this study with the rest of the world.

On the contrary to most of the available literature, in our setting, males were seen to perceive overall greater mistreatment compared to females [8], [10], [25]. This could be attributed to a greater percentage of perceived gender discrimination and due to cultural differences between other countries and Pakistan where females are protected and respected more. Another factor which can be taken into consideration is the difference in frequency of reported sexual harassment among men and women. Women have more frequently reported sexual harassment of all levels in the existing literature [26], [27].However; literature shows a similar frequency of reported belittlement and non-sexual harassment among the two genders in medical students [5].

Increased mistreatment was also observed in relation to a higher year of medical college which is supported by the cumulative effect of greater years spent in college, and also an increased level of sensitivity towards the perception of mistreatment. Our results in this respect parallel those of the earlier literature [8], [28]. This can also be explained on the basis that students in higher years of medical school experience an overall greater degree of perceived stress as reported earlier from Pakistan, India and Thailand [29], [30], [31].

The students reporting mistreatment also reported that the medical school does not have effective stress coping mechanisms for the students and does not help in coping with stress. This is a complaint which has been repeatedly mentioned and the need for better support systems has also been emphasized by earlier literature. This includes studies conducted both locally and internationally [14], [29], [32]. In order to cope with stress our medical students reported to have considered resorting to alcohol or drugs. This is similar to the results reported by some of the earlier studies on the coping strategies of medical students when under stress or tension [33]. However, earlier local study did not report alcohol or drugs as the one of the stress relievers stating sports, music, hanging out with friends, sleeping or going into isolation as the various coping mechanisms [29]. Also a study conducted in Nepal on medical students reported that alcohol/drug was the least used coping strategy [32].

Another consistent finding with other studies is the greater role of clinical faculty and residents as being the source of mistreatment as compared to nurses, patients, students and basic science faculty [4], [5]. This is partly explicable by greater one on one interactions and small group teaching methods. This can also be attributed to the fact that the consultants or senior doctors are the ones with the highest degree of authority in our set up of clinical rotations with almost no checks and balances, so they tend to misuse the given authority. The nurses on the other hand are also seen to be perpetrators of humiliation as they themselves are often mistreated by the senior doctors and they may vent this by humiliating either junior doctors or medical students. Role models, especially amongst medical staff, are very influential on students so this source of humiliation is particularly disappointing.

A difference in power, or perceived power, is required before humiliation can occur as pointed out earlier too. Therefore, sources of mistreatment of students have different hierarchies in the medical school and hence may have led to the difference in forms of mistreatment by faculty and residents. This is demonstrated by the results which show that stealing work or taking credit was mostly associated with residents whereas mistreatment from the clinical faculty was mostly in the form of being racially or religiously discriminated, shouted or yelled at, assigned extra tasks as punishment, threatened to fail or graded unfairly.

A secondary aspect of the study was to assess risk of Psychiatric morbidity among the medical students. This was found to be comparable to studies reported from both the developed and the developing world [14], [34], [35], [36], [37], [38], [39], [40]. In the present study, the 12-item GHQ was used with a cut off score of 3/4 providing the best conservative estimate of psychiatric morbidity [41]. Another study which was done to evaluate the validity and reliability of the GHQ 12 in Iran where the authors use the bimodal method for scoring also reports the mean GHQ 12 score to be 3.7 and recommend that this can be used as a cutoff for caseness in future studies [42]. Furthermore, a community based study in Japan indicates a mean GHQ 12 score of 3.6 [43]. Therefore, there is sufficient data to support the use of a cut off score of 3 in the present study for evaluation of psychiatric morbidity.

Earlier studies from United Kingdom using a cut off score of 3–4 report similar findings for prevalence of psychiatric morbidity [35], [38], [44]. Studies not using the same cut off score also report comparable estimates of psychiatric distress [36]. However, a study from Nepal using GHQ 12 and a cut off score of 4–5 suggested an overall prevalence of psychological morbidity of 20.9% [32]. There are also studies on stress which have either not used GHQ or used various other instruments for measuring the stress levels among the medical students. Sherina MS et al reported 41.9% of the medical students in Malaysia to have psychological stress [45]. Despite the variability of cut-offs and methods used to estimate the prevalence, psychiatric morbidity in our study can be considered comparable with earlier studies. These results reflect the general mental health and quality of life of medical students.

When compared to existing local figures of psychiatric morbidity, we found large differences. The point prevalence's of depression and anxiety in medical schools had higher figures probably overestimating the psychiatric morbidities [17], [18]. The students with a higher degree of psychiatric morbidity were also seen to be complaining about the medical school not providing adequate stress coping facilities. Psychiatric morbidity was also seen to be higher in clinical years as compared to non clinical years which can be explained on the basis of stress levels being higher in higher years of medical school.

Despite concomitant high prevalence of perceived mistreatment and psychiatric morbidity in medical students of our setting, the lack of association between the two entities highlights the two events to be independent. They are two separate issues which however can be dealt with similar interventions.

This study was the first to evaluate the mistreatment met by Pakistani medical students. Earlier studies have evaluated the levels of anxiety, depression and other psychological determinants and the factors associated with them but there has been no study focused on the belittlement of medical students. Also ours is the very first study aimed at finding a correlation between perceived mistreatment and psychological morbidity. The use of binary scoring method for the GHQ 12 scoring strengthens the results of the study further as it widely acceptable to be easily understandable by the general population apart from being robust to use and less challenging for the researchers as well [46], [47].

This was a cross sectional study giving a snapshot view of the medical student population and did not allow us to study the cause and effect relationship between psychiatric morbidity and the various numerous stressors. Further longitudinal studies assessing causal relationship between the factors identified in our study and perceived mistreatment and psychiatric morbidity will be helpful.

The presence of non response bias is another limitation of the study. Interviewing the non responders at a later time would be a worthwhile in future studies. The variability in GHQ 12 scoring methods and effect of cut off scores on results has always been a matter of controversy in such studies and so ours is no different from others. The scoring method recommended by Goldberg is the binary method. We used a cutoff score of 3 or more which is recommended by some studies on one hand but not encouraged by others. A study conducted by the WHO reports 1/2 to be the best cut off threshold for binary scoring [46].

Another limitation of this study was its confinement to the student population of a private medical university so the results cannot be entirely generalized for the rest of the medical students of Pakistan. Therefore, there is a need of conducting similar studies on a larger scale also in the public sector.

There are various coping mechanisms which can be used and should be promoted including mainly institution based support groups/systems involving both faculty and friends/classmates. Such measures should particularly focus hostilites as they cannot obtain support from family on a regular basis. Also seeking help from religion and other healthy activities such as sports, music and proper sleep are potential stress relieving methods.

Our study suggests a high prevalence of perceived mistreatment and psychiatric morbidity among medical students in a private medical institution in Karachi. However, there is no significant association between the two important hurdles of medical student life. More extensive, national surveys need to be conducted to obtain generalized results on which reforms to protect medical students can be formulated. Institution based support groups and mentorship programmes, particularly for hostelites may help reduce the level of stress and psychiatric morbidity. Medical institutions should invest in evidence based interventions to improve student life.

## Supporting Information

Table S1 Reported mistreatment.
(0.08 MB DOC)

Table S2 Psychiatric morbidity.
(0.09 MB DOC)

## References

[1] Nansel TR, Overpeck M, Pilla RS, Ruan WJ, Simons-Morton B (2001). Bullying behaviors among US youth: prevalence and association with psychosocial adjustment.. JAMA.

[2] Jorgenson LM, Wahl KM (2000). Workplace Sexual Harassment: Incidence, Legal Analysis, and the Role of the Psychiatrist.. Harvard Review of Psychiatry.

[3] Krieger N, Waterman PD, Hartman C, Bates LM, Stoddard AM (2006). Social hazards on the job: workplace abuse, sexual harassment, and racial discrimination-A study of Black, Latino and White Low-Income women and men workers in the United States.. International Journal of Health Services.

[4] Baldwin DC, Daugherty SR, Eckenfels EJ (1991). Student perceptions of mistreatment and harassment during medical school. A survey of ten United States schools.. West J Med.

[5] Frank E, Carrera JS, Stratton T, Bickel J, Nora LM (2006). Experiences of belittlement and harassment and their correlates among medical students in the United States: longitudinal survey.. Bmj.

[6] Lebenthal A, Kaiserman I, Lernau O (1996). Student abuse in medical school: a comparison of students' and faculty's perceptions.. Isr J Med Sci.

[7] Mangus RS, Hawkins CE, Miller MJ (1998). Prevalence of harassment and discrimination among 1996 medical school graduates: a survey of eight US schools.. Jama.

[8] Rautio A, Sunnari V, Nuutinen M, Laitala M (2005). Mistreatment of university students most common during medical studies.. BMC Med Educ.

[9] Uhari M, Kokkonen J, Nuutinen M, Vainionpaa L, Rantala H (1994). Medical student abuse: an international phenomenon.. Jama.

[10] Nora LM, McLaughlin MA, Fosson SE, Stratton TD, Murphy-Spencer A (2002). Gender discrimination and sexual harassment in medical education: perspectives gained by a 14-school study.. Acad Med.

[11] Frank E, Carrera JS, Elon L, Hertzberg VS (2006). Basic demographics, health practices, and health status of U.S. medical students.. Am J Prev Med.

[12] Benitez MH, de las Cuevas Castresana C, Rodriguez Pulido F, Garcia-Estrada Perez A, Gonzalez de Rivera Revuelta JL (1989). [A comparative psychopathologic study of university students].. Actas Luso Esp Neurol Psiquiatr Cienc Afines.

[13] Dyrbye LN, Thomas MR, Shanafelt TD (2006). Systematic review of depression, anxiety, and other indicators of psychological distress among U.S. and Canadian medical students.. Acad Med.

[14] Dahlin ME, Runeson B (2007). Burnout and psychiatric morbidity among medical students entering clinical training: a three year prospective questionnaire and interview-based study.. BMC Med Educ.

[15] Aktekin M, Karaman T, Senol YY, Erdem S, Erengin H (2001). Anxiety, depression and stressful life events among medical students: a prospective study in Antalya, Turkey.. Med Educ.

[16] Tyssen R, Vaglum P, Gronvold NT, Ekeberg O (2001). Factors in medical school that predict postgraduate mental health problems in need of treatment. A nationwide and longitudinal study.. Med Educ.

[17] Inam SN, Saqib A, Alam E (2003). Prevalence of anxiety and depression among medical students of private university.. J Pak Med Assoc.

[18] Khan MS, Mahmood S, Badshah A, Ali SU, Jamal Y (2006). Prevalence of depression, anxiety and their associated factors among medical students in Karachi, Pakistan.. J Pak Med Assoc.

[19] Makowska Z, Merecz D, Moscicka A, Kolasa W (2002). The validity of general health questionnaires, GHQ-12 and GHQ-28, in mental health studies of working people.. Int J Occup Med Environ Health.

[20] Muhamad SBY, Ahmad FAR, Yaacob MJ (2010). The sensitivity, specificity and reliability of the Malay version 12-items General Health Questionnaire (GHQ-12) in detecting distressed medical students.. ASEAN Journal of Psychiatry.

[21] Piccinelli M, Bisoffi G, Bon MG, Cunico L, Tansella M (1993). Validity and test-retest reliability of the Italian version of the 12-item General Health Questionnaire in general practice: a comparison between three scoring methods.. Compr Psychiatry.

[22] Maida AM, Vasquez A, Herskovic V, Calderon JL, Jacard M (2003). A report on student abuse during medical training.. Med Teach.

[23] Nagata-Kobayashi S, Sekimoto M, Koyama H, Yamamoto W, Goto E (2006). Medical student abuse during clinical clerkships in Japan.. J Gen Intern Med.

[24] Silver HK, Glicken AD (1990). Medical student abuse. Incidence, severity, and significance.. JAMA.

[25] Larsson C, Hensing G, Allebeck P (2003). Sexual and gender-related harassment in medical education and research training: results from a Swedish survey.. Med Educ.

[26] Witte FM, Stratton TD, Nora LM (2006). Stories from the field: students' descriptions of gender discrimination and sexual harassment during medical school.. Acad Med.

[27] Moscarello R, Margittai KJ, Rossi M (1994). Differences in abuse reported by female and male Canadian medical students.. CMAJ.

[28] Richardson DA, Becker M, Frank RR, Sokol RJ (1997). Assessing medical students' perceptions of mistreatment in their second and third years.. Acad Med.

[29] Shaikh BT, Kahloon A, Kazmi M, Khalid H, Nawaz K (2004). Students, stress and coping strategies: a case of Pakistani medical school.. Educ Health (Abingdon).

[30] Supe AN (1998). A study of stress in medical students at Seth G.S. Medical College.. J Postgrad Med.

[31] Saipanish R (2003). Stress among medical students in a Thai medical school.. Med Teach.

[32] Sreeramareddy CT, Shankar PR, Binu VS, Mukhopadhyay C, Ray B (2007). Psychological morbidity, sources of stress and coping strategies among undergraduate medical students of Nepal.. BMC Med Educ.

[33] Tyssen R, Vaglum P, Aasland OG, Gronvold NT, Ekeberg O (1998). Use of alcohol to cope with tension, and its relation to gender, years in medical school and hazardous drinking: a study of two nation-wide Norwegian samples of medical students.. Addiction.

[34] Assadi SM, Nakhaei MR, Najafi F, Fazel S (2007). Mental health in three generations of Iranian medical students and doctors. A cross-sectional study.. Soc Psychiatry Psychiatr Epidemiol.

[35] Guthrie E, Black D, Bagalkote H, Shaw C, Campbell M (1998). Psychological stress and burnout in medical students: a five-year prospective longitudinal study.. J R Soc Med.

[36] Benitez C, Quintero J, Torres R (2001). [Prevalence of risk for mental disorders among undergraduate medical students at the Medical School of the Catholic University of Chile].. Rev Med Chil.

[37] Carson AJ, Dias S, Johnston A, McLoughlin MA, O'Connor M (2000). Mental health in medical students. A case control study using the 60 item General Health Questionnaire.. Scott Med J.

[38] Guthrie EA, Black D, Shaw CM, Hamilton J, Creed FH (1995). Embarking upon a medical career: psychological morbidity in first year medical students.. Med Educ.

[39] Willcock SM, Daly MG, Tennant CC, Allard BJ (2004). Burnout and psychiatric morbidity in new medical graduates.. Med J Aust.

[40] Shariati M, Yunesian M, Vash JH (2007). Mental health of medical students: a cross-sectional study in Tehran.. Psychol Rep.

[41] Mario M WG, Juan-Jose L, Norman S (2002). Psychiatric diagnosis and classification. 3rd edition ed.

[42] Montazeri A, Harirchi AM, Shariati M, Garmaroudi G, Ebadi M (2003). The 12-item General Health Questionnaire (GHQ-12): translation and validation study of the Iranian version.. Health Qual Life Outcomes.

[43] Toyabe S, Shioiri T, Kobayashi K, Kuwabara H, Koizumi M (2007). Factor structure of the General Health Questionnaire (GHQ-12) in subjects who had suffered from the 2004 Niigata-Chuetsu Earthquake in Japan: a community-based study.. BMC Public Health.

[44] Firth J (1986). Levels and sources of stress in medical students.. Br Med J (Clin Res Ed).

[45] Sherina MS, Rampal L, Kaneson N (2004). Psychological stress among undergraduate medical students.. Med J Malaysia.

[46] Goldberg DP, Gater R, Sartorius N, Ustun TB, Piccinelli M (1997). The validity of two versions of the GHQ in the WHO study of mental illness in general health care.. Psychol Med.

[47] Chipimo PJ, Fylkesnes K (2010). Comparative validity of screening instruments for mental distress in zambia.. Clin Pract Epidemiol Ment Health.

