# Supplementary material for: Prevalence of Mistreatment or Belittlement among Medical Students – A Cross Sectional Survey at a Private Medical School in Karachi, Pakistan
Source: PLoS One. 2010 Oct 15;5(10):e13429. doi: 10.1371/journal.pone.0013429 (PMC2955546; doi:10.1371/journal.pone.0013429)
Supplement: Table S1 — Reported mistreatment. (0.08 MB DOC) [file pone.0013429.s001.doc]

Table S1: Reported Mistreatment

|  | No  % (n) | Yes  % (n) | Total  % (n) | *p*-value |
| --- | --- | --- | --- | --- |
| **Gender** | | | | |
| **Male**  **Female** | 30.3(36) | 69.7(83) | 51.3(119) | 0.019 |
|  | 45.1(51) | 54.9(62) | 48.7(113) |  |
| **Religious Identity** | | | | |
| **Very Strong**  **Strong**  **Moderate**  **Low**  **None** | 26.8 (11) | 73.2 (30) | 17.7(41) | 0.298 |
|  | 38.8(38) | 61.2(60) | 42.2(98) |  |
|  | 43.0(34) | 57.0(45) | 34.1(79) |  |
|  | 36.4(4) | 63.6(7) | 4.7(11) |  |
|  | 0(0) | 100.0(3) | 1.3(3) |  |
| **Marital Status** | | | | |
| **Single**  **Married** | 38.3(85) | 61.7(137) | 95.7(222) | 0.266 |
|  | 20.0(2) | 80.0(8) | 4.3(10) |  |
| **Parents or close relative a physician** | | | | |
| **No**  **Yes** | 35.3(36) | 64.7(66) | 44.0(102) | 0.539 |
|  | 39.2(51) | 60.8(79) | 56.0(130) |  |
| **Ethinic group** | | | | |
| **Punjabi**  **Pathan**  **Sindhi**  **Balouchi**  **Urdu Speaking**  **Other** | 36.4(36) | 63.6(63) | 42.7(99) | 0.801 |
|  | 31.2(10) | 68.8(22) | 13.8(32) |  |
|  | 43.8(7) | 56.2(9) | 6.9(16) |  |
|  | 25.0(1) | 75.0(3) | 1.7(4) |  |
|  | 36.7(18) | 63.3(31) | 21.1(49) |  |
|  | 46.9(15) | 53.1(17) | 13.8(32) |  |
| **Geographical Background** | | | | |
| **Rural**  **Urban** | 37.3(19) | 62.7(32) | 22.0(51) | 0.967 |
|  | 37.6(68) | 62.4(113) | 78.0(181) |  |
| **Monthly household income** | | | | |
| **10,000-50,000**  **50,000-100,000**  **>100,000** | 28.8(15) | 71.2(37) | 23.5(52) | 0.089 |
|  | 45.9(39) | 54.1(46) | 38.5 (85) |  |
|  | 33.3(28) | 66.7(56) | 38.0(84) |  |
| **Ever failed a rotation**  **(continuous assessment)?** | | | | |
| **No**  **Yes** | 19.4(6) | 80.6(25) | 14.0(31) | 0.029 |
|  | 39.8(76) | 60.2(115) | 86.0(191) |  |
| **Year division** | | | | |
| **Pre-clinical**  **Clinical** | 48.6(35) | 51.4(37) | 31.0(72) | 0.019 |
|  | 32.5(52) | 67.5(108) | 69.0(160) |  |
| **Psychiatric Morbidity** | | | | |
| **Healthy**  **Morbid** | 42.4(61) | 57.6(83) | 65.2(144) | 0.068 |
|  | 29.9(23) | 70.1 (54) | 34.8(77) |  |
| **My medical school tries to**  **minimize stress** | | | | |
| **Agree**  **Neutral**  **Disagree** | 48.6(17) | 51.4(18) | 15.5(35) | 0.009 |
|  | 55.9(19) | 44.1(15) | 15.0(34) |  |
|  | 31.2(49) | 68.8(108) | 69.5(157) |  |
| **My medical school has a good**  **system to help student cope with stress** | | | | |
| **Agree**  **Neutral**  **Disagree** | 45.5(10) | 54.5(12) | 9.7(22) | 0.032 |
|  | 53.7(22) | 46.3(19) | 18.1(41) |  |
|  | 32.5(53) | 67.5(110) | 72.1(163) |  |
| **I started/may start smoking**  **to cope with stress in medical school** | | | | |
| **Agree**  **Neutral**  **Disagree** | 36.0(9) | 64.0(16) | 11.1(25) | 0.564 |
|  | 26.3(5) | 73.7(14) | 8.4(19) |  |
|  | 38.7(70) | 61.3(111) | 80.4(181) |  |
| **I may consider use of alcohol/**  **drugs to cope with stress in medical school** | | | | |
| **Agree**  **Neutral**  **Disagree** | 25.0(7) | 75.0(21) | 12.4(28) | 0.023 |
|  | 17.4(4) | 82.6(19) | 10.2(23) |  |
|  | 42.3(74) | 57.7(101) | 77.4(175) |  |
