# Supplementary material for: Prevalence of Mistreatment or Belittlement among Medical Students – A Cross Sectional Survey at a Private Medical School in Karachi, Pakistan
Source: PLoS One. 2010 Oct 15;5(10):e13429. doi: 10.1371/journal.pone.0013429 (PMC2955546; doi:10.1371/journal.pone.0013429)
Supplement: Table S2 — Psychiatric morbidity. (0.09 MB DOC) [file pone.0013429.s002.doc]

Table S2: Psychiatric Morbidity

|  | Healthy  % (n) | | Morbid  % (n) | Total  % (n) | *p*-value |
| --- | --- | --- | --- | --- | --- |
| **Gender** | | | | | |
| **Male**  **Female** | 55.6(80) | | 45.5(35) | 52.0(115) | 0.152 |
|  | 44.4 (64) | | 54.5(42) | 48.0(106) |  |
| **Religious Identity** | | | | | |
| **Very Strong**  **Strong**  **Moderate**  **Low**  **None** | 18.8(27) | | 18.2(14) | 18.6(41) |  |
|  | 43.8(63) | | 40.3(31) | 42.5(94) |  |
|  | 29.9(43) | | 37.7(29) | 32.6(72) |  |
|  | 5.6(8) | | 3.9(3) | 5.0(11) |  |
|  | 2.1(3) | | .0(0) | 1.4(3) |  |
| **Marital Status** | | | | | |
| **Single**  **Married** | 93.8(135) | | 98.7(76) | 95.5(211) | 0.171 |
|  | 6.2(9) | | 1.3(1) | 4.5(10) |  |
| **Parents or close relative a physician** | | | | | |
| **No**  **Yes** | 42.4(61) | | 48.1(37) | 44.3(98) | 0.417 |
|  | 57.6(83) | | 51.9(40) | 55.7(123) |  |
| **Ethinic group** | | | | |  |
| **Punjabi**  **Pathan**  **Sindhi**  **Balouchi**  **Urdu Speaking**  **Other** | 44.4(64) | | 39.0(30) | 42.5(94) |  |
|  | 11.8(17) | | 16.9(3) | 13.6(30) |  |
|  | 7.6(11) | | 6.5(5) | 7.2(16) |  |
|  | 1.4(2) | | 2.6(2) | 1.8(4) |  |
|  | 18.8(27) | | 23.4(18) | 20.4(45) |  |
|  | 16.0(23) | | 11.7(9) | 14.5(32) |  |
| **Geographical Background** | | | | | |
| **Rural**  **Urban** | 25.7(37) | | 14.3(11) | 21.7(48) | 0.050 |
|  | 74.3(107) | | 85.7(66) | 78.3(173) |  |
| **Monthly household income** | | | | | |
| **10,000-50,000**  **50,000-100,000**  **>100,000** | 20.1(27) | | 28.9(22) | 23.3(49) | 0.181 |
|  | 38.1(51) | | 40.8(31) | 39.0(82) |  |
|  | 41.8(56) | | 30.3(23) | 37.6(79) |  |
| **Ever failed a rotation**  **(Continuous assessment)?** | | | | | |
| **No**  **Yes** | 15.3(21) | | 12.2(9) | 14.2(30) | 0.530 |
|  | 84.7(116) | | 87.8(65) | 85.8(181) |  |
| **Year division** | | | | | |
| **Pre-clinical**  **Clinical** | 26.4(38) | | 39.0(30) | 30.8(68) | 0.054 |
|  | 73.6(106) | | 61.0(47) | 69.2(153) |  |
| **How much does this mistreatment**  **Bother you?** | | | | | |
| **A lot**  **Not at all**  **Does not apply** | | 57.6(83) | 74.0(57) | 63.3(140) | 0.049 |
|  |  | 6.2(9) | 2.6(2) | 5.0(11) |  |
|  |  | 36.1(52) | 23.4(18) | 31.7(70) |  |
| **How often does this mistreatment**  **occur at your university?** | | | | | |
| **Never**  **Rarely**  **Often** | | 33.3(48) | 26.0(20) | 30.8(68) | 0.206 |
|  |  | 12.5(18) | 7.8(6) | 10.9(24) |  |
|  |  | 54.2(78) | 66.2(51) | 58.4(129) |  |
| **My medical school tries to**  **minimize stress** | | | | | |
| **Agree**  **Neutral**  **Disagree** | | 16.9(24) | 14.3(11) | 16.0(35) | 0.323 |
|  |  | 16.9(24) | 10.4(8) | 14.6(32) |  |
|  |  | 66.2(94) | 75.3(58) | 69.4(152) |  |
| **My medical school has a good**  **system to help student cope with stress** | | | | | |
| **Agree**  **Neutral**  **Disagree** | | 12.0(17) | 6.5(5) | 10.0(22) | 0.011 |
|  |  | 22.5(32) | 9.1(7) | 17.8(39) |  |
|  |  | 65.5(93) | 84.4(65) | 72.1(158) |  |
| **I started/may start smoking to cope**  **with stress in medical school** | | | | | |
| **Agree**  **Neutral**  **Disagree** | | 8.5(12) | 16.9(13) | 11.5(25) | 0.179 |
|  |  | 8.5(12) | 7.8(6) | 8.3(18) |  |
|  |  | 83.0(117) | 75.3(58) | 80.3(175) |  |
| **I may consider use of alcohol/ drugs**  **to cope with stress in medical school** | | | | | |
| **Agree**  **Neutral**  **Disagree** | | 10.6(15) | 16.9(13) | 12.8(28) | 0.331 |
|  |  | 11.3(16) | 7.8(6) | 10.0(22) |  |
|  |  | 78.2(111) | 75.3(58) | 77.2(169) |  |
